# Evaluation of the resection plane three-dimensional positional accuracy using a resection guide directional guidance slot; a randomized clinical trial

**DOI:** 10.1186/s12903-024-04476-3

**Published:** 2024-06-27

**Authors:** Yehia El-Mahallawy, Noha Y. Dessoky, Hams H. Abdelrahman, Haytham Al-Mahalawy

**Affiliations:** 1 Oral and Maxillofacial Surgery Department, Faculty of Dentistry, Alexandria University, Alexandria, Egypt; 2 Dental Public Health and Pediatric Dentistry Department, Faculty of Dentistry, Alexandria University, Alexandria, Egypt; 3 Oral and Maxillofacial Surgery Department, Faculty of Dentistry, Fayoum University, Fayoum, Egypt

**Keywords:** Mandibular resection, Surgery, Computer-assisted, Computer‐aided design, Computer‐aided manufacturing, Data accuracy, Software

## Abstract

**Aim:**

The study was performed to compare the mandibular resection guide with a directional guidance slot with the conventional guide regarding three-dimensional positional accuracy.

**Materials and methods:**

Twenty-six patients with lateral segmental mandibular defects were selected, and randomly allocated into two groups. All defects were managed with preoperative virtual surgical planning. Resection in the test group was conducted using a resection guide with a directional guidance slot, while a conventional resection guide design was utilized in the control group. The linear and angular deviation of the osteotomy planes was analyzed for both groups, along with the accuracy of the insertion of the reconstruction bone block in the resected defect. Data were documented, absolute deviation was calculated, statistical analysis was performed and significance was set at the 5% level.

**Results:**

The cases conducted with a directional guidance templet reported a statistically significant difference when compared to the conventional edge-cutting guide regarding the linear and angular spatial osteotomy plane position (*P* < 0.001). The defect span analysis reported excellent levels of agreement in both groups (ICC = 1.00, ICC = 0.995), however, the difference between the groups was statistically significant (*P* < 0.001).

**Conclusion:**

The study demonstrated the enhanced positional accuracy of the resection plane and reconstruction block placement when a directional slot is incorporated in the computer-generated resection guide.

## Introduction

Patients with malignant and benign disease abutting or invading the mandible often undergo segmental resection of the mandible. To restore continuity of the mandible and associated function and aesthetics, reconstruction with a titanium plate in combination with an osseous free flap is performed in the majority of cases [1, 2]. The resection planes of the involved part of the mandible must be determined accurately to ensure adequate and free margins, but also to allow precise placement of bone segments, enabling the contour of the neo-mandible to match the native resected mandible [1, 2].

The exact location of the resection planes, as well as the reconstruction after resection, can be prepared with Virtual Surgical Planning (VSP) [3]. Using this technique, Three-Dimensional (3D) rendered models of the mandible and graft are constructed from a preoperative computed tomography scan. The 3D models are used to perform a virtual (segmental) mandibulectomy and to virtually segment the graft to match the defect. To translate the position of the resection planes from the virtual surgical plan to the clinical situation in the operating room, patient-specific cutting guides and fixation plates are designed and manufactured using Computer-Aided Design/Computer-Aided Manufacturing (CAD/CAM) techniques. These cutting guides enable the surgeon to perform the surgical procedure more accurately, while significantly shortening the operating time [4–6].

Postoperative Computed Tomography (CT) imaging has been used to verify how precise the virtual surgical plan has been translated during surgery. Several studies have evaluated the accuracy of the translation by comparing the location and orientation of the planned resection plane with the plane of the actual osteotomy performed. El-Mahallawy et al. introduced a landmark-based postoperative accuracy assessment methodology to get the deviation of the actual postoperative outcome from the virtual plan [7]. De Maesschalck et al. and Roser et al. used a slightly different method and measured the maximum distance between the planned and actual resection planes, rather than landmarks [8, 9]. Mean deviations of 2–2.3 mm between the preoperatively planned and postoperative resection planes were reported [7–9].

Previous studies regarding the accuracy of the resection plane only assess the accuracy of the translation of the preoperative plan to the intraoperative situation. The three-dimensional positional accuracy of the resection plane was introduced by Brouwer de Koning et al. [10].

The literature lacks a consensus regarding the ideal design of the resection guide. De Maesschalck et al. and Roser et al. utilized either a bony stump edge or lesion edge cutting ledge, while Kraeima et al. utilized a slot in the resection guide to direct the cutting device all the way through the thickness of the mandible [8, 9, 11]. Owing to the variation in the resection guide design, there is a need for a resection plane positional accuracy evlaution in order to aid in the selection of the most accurate design of the guide.

The study was designed to analyze the three-dimensional positional accuracy of the preoperatively planned mandibular resection osteotomy planes using different resection guide configurations. The null hypothesis of the current study was that the utilization of a resection guide for segmental mandibular resection with a directional guidance slot will yield a superior three-dimensional positional accuracy of the resection plane than the conventional resection guide design. The specific aims were to 1) compare the linear and angular deviation of the postoperative osteotomy planes conducted with a resection guide with a directional guidance slot with the conventional guide, 2) evaluate the insertion accuracy of the harvested reconstruction bone block, and 3) analysis of the guide’s ability in maintaining the spatial relation after resection by defect span analysis.

## Materials and methods

### Study design

The positional accuracy of the mandibular resection osteotomy planes using different resection guide configurations was appraised in a Parallel, Randomized Clinical Trial with accordance to the CONSORT guidelines (http://www.consort-statement.org) [12]. Sample size analysis was performed using the Mann–Whitney test with a 5% level of significance, 80% power, and adding 10% loss to follow up (G*power, 3.1.9.4) [13]. It was estimated that a minimum of 26 patients undergoing segmental mandibular resection, 13 per group, are required to detect an assumed difference of 5.5° in mean yaw rotation between directional and conventional resection guide with assumed groups standard deviation of (1.5, 6) respectively [10].

Patients with segmental mandibular continuity defect, not involving the condyle were enrolled in this study. They may be planned for either immediate (primary) or delayed (secondary) reconstruction. Patients were recruited from those admitted to the Outpatient Clinic of Alexandria University Teaching Hospital from December 2022 to January 2024. Patients with an active infection at the site of resection were excluded from the study. The declaration of Helsinki's ethical guidelines was considered during the conceptualization and conduction of this study. All patients signed an informed consent before the operation and were informed and accepted the nature of the study. Ethical committee approval was attained (IRB:00010556-IORG:0008830–0771-09/2023) and clinical trial registration was performed [PACTR202402846281250-(02/02/2024)]. Computer-generated randomization was conducted using 2 & 4 random block sizes, and 1:1 allocation (http://www.randomizer.org/). The randomization and group allocation process was executed by a distinct investigator (HA) not involved with the surgical team. Allocation concealment was conducted using an on-site computer system, where allocations are kept in a locked electronic file with only access to the surgical team.

### Preoperative virtual surgical planning

All of the enrolled patients in both groups underwent the same VSP protocol [7]. preoperative data acquisition was performed using a Multi-Detector Computed Tomography (MDCT) scan with a slice thickness of 0.6mm (Philips Brilliance 64 MDCT, Philips, Eindhoven, Netherlands).

The Computer-Assisted Surgery (CAS) protocol was implemented for all of the enrolled cases. All patients were treated at the Maxillofacial Unit of Alexandria University Hospital, Egypt. VSP commenced with radiographic examination with MDCT scan (Philips Brilliance 64 MDCT, Philips, Eindhoven, Netherlands) for the maxillofacial (slice thickness 0.6 mm) as well as the donor site (slice thickness 1.0 mm). Based on the predetermined treatment plan, a donor site radiograph was obtained. Digital Imaging and Communications in Medicine (DICOM) data were fed to a segmentation software (Mimics, Materialise, Leuven, Belgium) for bone tissue thresholding and 3D bone model conception. In the segmented mandible file, the outline of the lesion was marked along with the safety margin according to the nature of each causative factor. The resection margins and localization of the optimal angles for the mandibular osteotomies were determined to produce the Mandible *Resection-Osteotomy Guides* using the Computer-designing software (3Matic; Materialise).

In all of the enrolled patients, the Resection-Osteotomy Guide was designed twice, once in a conventional edge-cutting manner, and once with a directional guidance slot. The directional slot in the Resection-Osteotomy Guide dimensions was designed based on the thickness of the oscillating saw that is used in the resection (System 8 Precision, Stryker, Air-view Boulevard, Kalamazoo, MI, USA). A mirroring tool was used to create a New-Mandible Model with a symmetrical shape without the occurrence of the lesion irregularities on the affected side, and the 3D spatial relation between the proximal and distal segments after lesion virtual resection was maintained by a *Reconstruction-Fixation Template* creation to transfer this relation into the operation room. Both resection and fixation guides have the same screw-bore-hole position. Either the neo-mandible was printed for preoperative reconstruction plate adaptation or a virtually created plate was designed and printed according to each case treatment plan.

For each case and according to its assigned treatment plan the neo-mandible was superimposed on the donor site bone and a symmetrical final *Virtual Preoperative Model* (VPM) was created. All of the designed parts were exported to a 3D-printing software in a Standard Tessellation Language (STL) format (NETFAB, Autodesk, CA, USA). Fused Deposition Modeling (FDM) 3D printing of the guides was performed and they were sterilized following the Center for Disease Control guidelines (CDC) [14].

### Surgical procedure

For both groups and cases planned for primary reconstruction, the surgical procedure was conducted using a two-team approach. The surgical teams were unchangeable throughout the study. To avoid selection bias, the surgical team opened the assigned electronic file on the day of the operation for group allocation. For cases in the Test Group, a *Directional Guidance Slot Resection-Osteotomy Guide* was utilized, while for those in the Control Group a *Conventional Edge-Cutting Resection-Osteotomy Guide* was applied.

A second neck crease cervicectomy approach was used for mandible exposure. In the study group, the mandible Resection-Osteotomy Guide with the directional guidance slot was fixed in place using a 2.0 mm screw, followed by lesion resection through the slot using an oscillating saw. On the other hand, resection in the control group was conducted by guide edge cutting. For both groups, the Reconstruction-Fixation Template was fixed while the occlusion is checked to ensure an adequate position of the proximal segment after resection, and the fixation device was implanted with a minimum of three screws in each stump. For those planning for primary reconstruction, the harvested graft/flap was inserted in the defect position and fixed with the reconstruction plate.

### Virtual planning accuracy analysis.

**Fig. 1:**
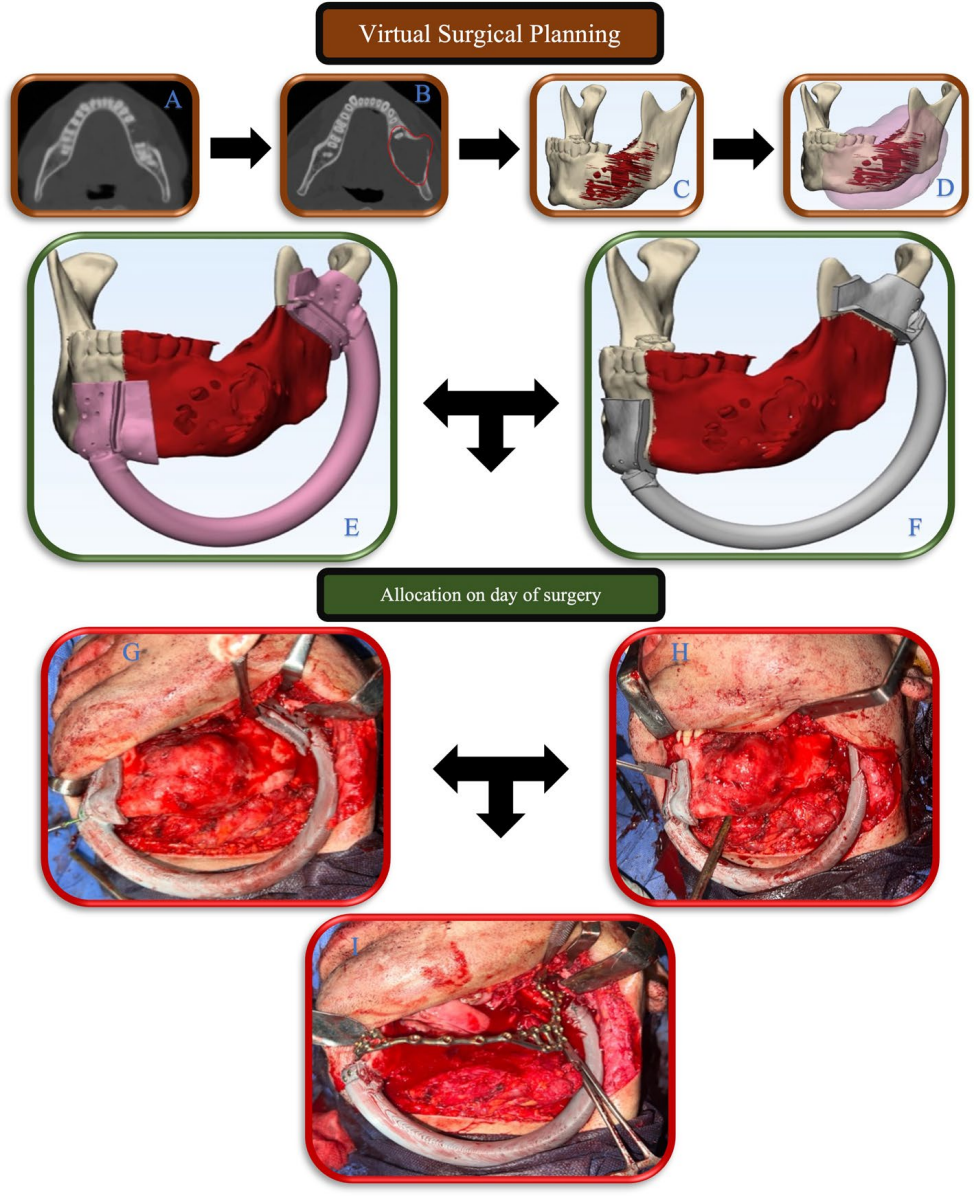
Flow chart of the virtual planning procedure. Obtaining of DICOM data and lesion outline (**A**, **B**), Followed by thresholding and bone model creation (**C**). the safety margin is determined according to the nature of the disease (**D**). The Resection-Osteotomy Guide is designed with tow different configurations, a guide with a directional guidance slot (**E**) and a conventional edge-cutting guide (**F**). according to the randomization processes, choice of the utilized guide was allocated on the day of the surgery. This was followed by Guide insertion (**G**), resection (**H**), and fixation (**I**)

Owing to the fact that virtual planning was conducted in both groups, the outcome assessor (HA) was masked from which type of guide was utilized in each case. An immediate postoperative MSCT scan was obtained within 7 days of the surgery using the same preoperative scanning parameters. The postoperative MSCT DICOM data was segmented to isolate the reconstructed mandible and to create an *Actual Postoperative Model* (APM). The postoperative accuracy of the virtually assisted surgery was conducted in accordance with the standard methodology proposed by El‐Mahallawy et al. and van Baar et al. using the 3D-analysis software (GOM Inspect Pro 2019, GmbH, Braunschweig, Germany) [7, 15]. For each of the selected 2D and 3D parameters, the deviation was calculated by subtracting the APM values from the VPM. The absolute mean (Δ) for all of the operated patients was calculated. In both models, the resected part, the reconstruction blocks, and the remaining stumps were segmented and separated for better analysis of the resection plane (Fig. 1). Resection plane assignment was performed manually using the 3-Point Plane tool on the GOM Inspect Pro software. Two of three assigned points were at the lower border of the resection stumps, one buccal and the other lingual. The remaining point was assigned at the uppermost bony border of each of the remaining stumps.

#### Guide positional accuracy analysis

**Fig. 2:**
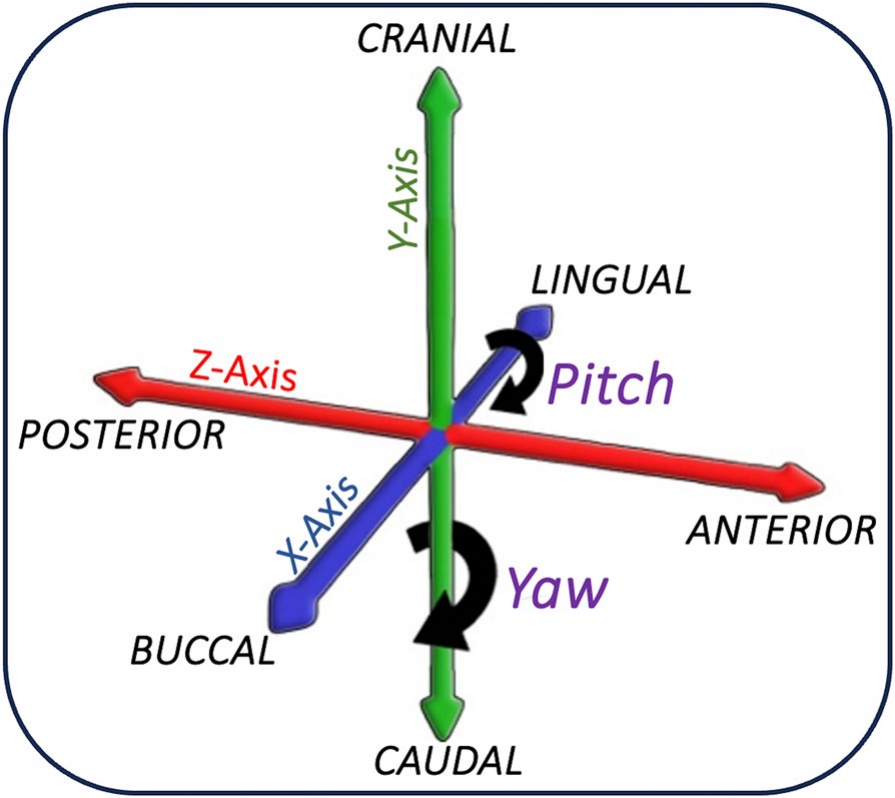
Descriptive illustration for the angular resection plane osteotomy. The pitch angle is defined as the rotation around the Bucco-Lingual axis. The yaw angle is defined as the rotation around the Cranio-Caudal axis. Since the resection was conducted along the Antero-Posterior plane, there is no need for the analysis of the roll angle

**Fig. 3:**
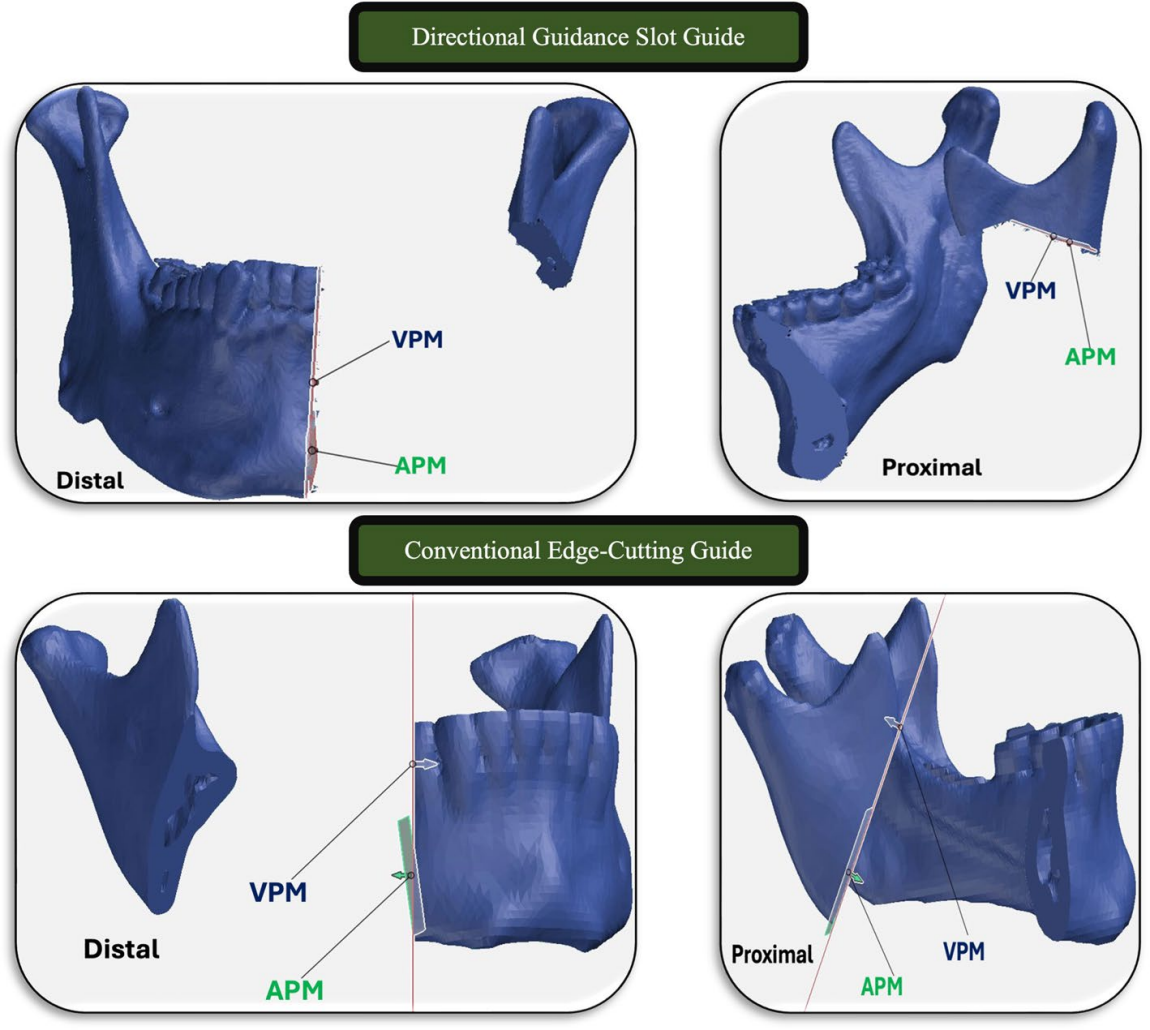
Analysis of the Mandible Resection-Osteotomy Guides accuracy for cases in both groups. The virtual (red) and the actual (Green) planes were superimposed and linear and angular differences was calculated

The actual position of each osteotomy was compared with its virtual counterpart, and the difference was presented in mm. For linear deviation analysis, the distance difference between the actual and virtual osteotomies was calculated in both the proximal and distal osteotomies. For angular deviation analysis of the proximal and distal osteotomies, the pitch and Yaw rotation were analyzed. The osteotomy plane *Roll-Rotation* around the anteroposterior axis won’t be evaluated as a full thickness resection is conducted from the buccal cortex up to its lingual counterpart. Figure 2 defines each rotation with the corresponding axis. *Pitch-Rotation* was determined by calculating the degree of plane rotation around the buccolingual axis, while *Yaw-Rotation* was determined by calculating the degree of plane rotation around the craniocaudal axis Figure 3.

#### Flap/ graft positional accuracy analysis

**Fig. 4:**
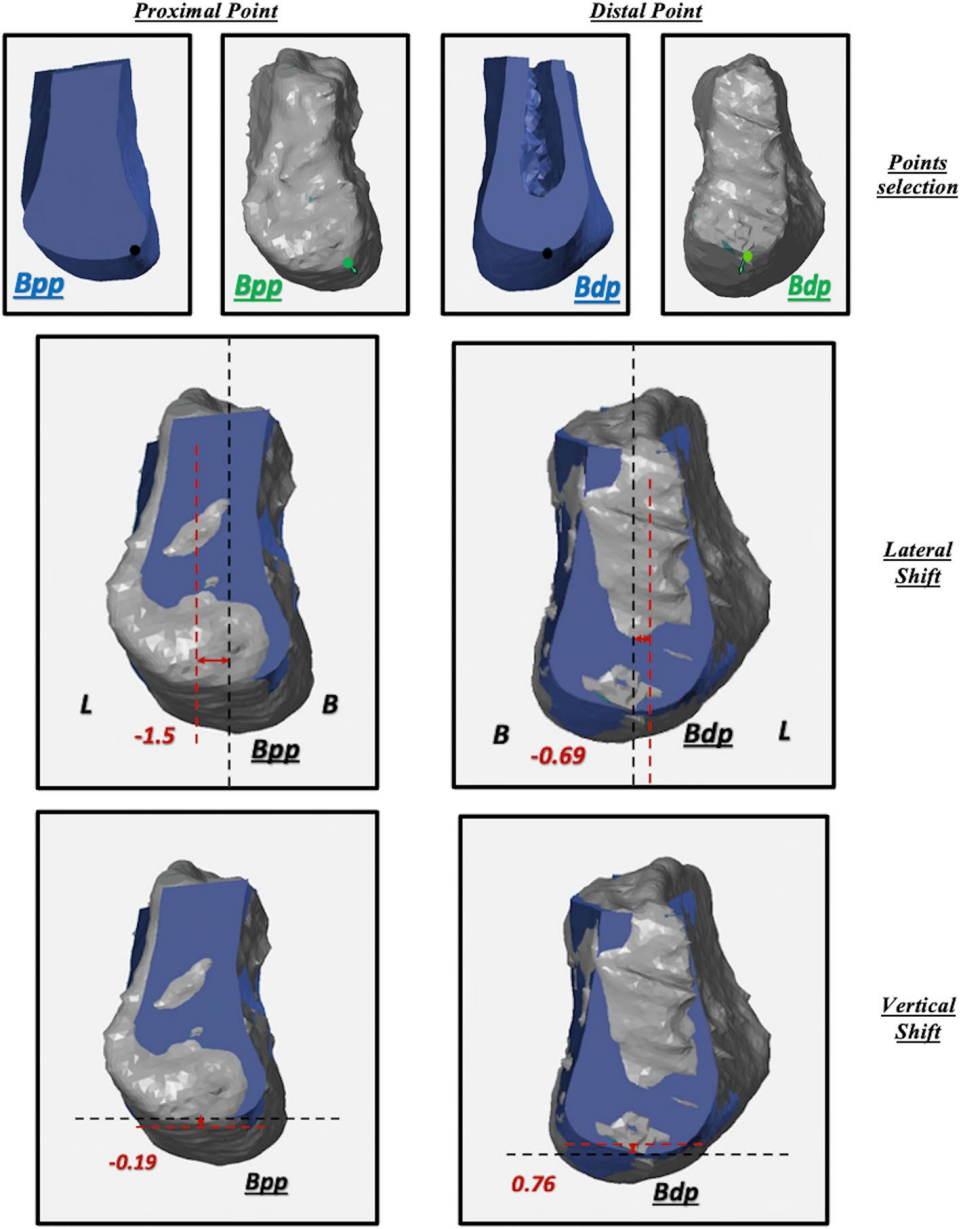
Analysis of the harvested reconstruction bone block position. The VPM plane is represented by the black dotted line, while the APM plane is represented by the red dotted line. The “ + ” sign was annotated to deviation in the cranial or buccal directions, while the “-” sign was given to deviations in the caudal or lingual direction

Insertion Accuracy of the harvested reconstruction bone block in the resected defect was evaluated by calculating the lateral and vertical block shift for both the proximal and distal flap/ graft block points (Bpp and Bdp). Lateral Block Shift was calculated between the bloc points and the Mid-sagittal Plane (MSP), while Vertical Block Shift was calculated between the block points and the Frankfurt Horizontal Plane (FHP) Figure 4.

#### Defect span accuracy analysis

Defect span analysis was utilized to further examine the guide's ability to maintain the spatial relation after resection. The distance between the proximal and the distal plane was determined in the VPM, which was then compared to the value obtained in the APM.

### Statistical analysis

Data was analyzed using the IBM SPSS for Windows V.23.0. (IBM Corp, NY, USA). The significance of the obtained results was judged at the 5% level, and data was presented in absolute mean, standard deviation, and range. Data normality was tested using the Shapiro–Wilk test, where the Student t-test was used to compare normally distributed variables and the Mann Whitney test was utilized with non-normally distributed ones. Agreement between the VPM and the APM defect span values were compared using the Intra-class Correlation coefficient (ICC) [7, 16].

## Results

The study was conducted on 26 patients with lateral mandibular defects, where 13 patients in each group received the intervention and were analysed. The mean reported age was 37.7 ± 12.6 years with a male-to-female ratio of 0.73:1. A patient-specific reconstruction plate was used in 9 cases, while fixation was attained in the remaining cases using a preoperatively adapted reconstruction plate. The patient’s demographic data is documented in Table 1.

**Table 1:**
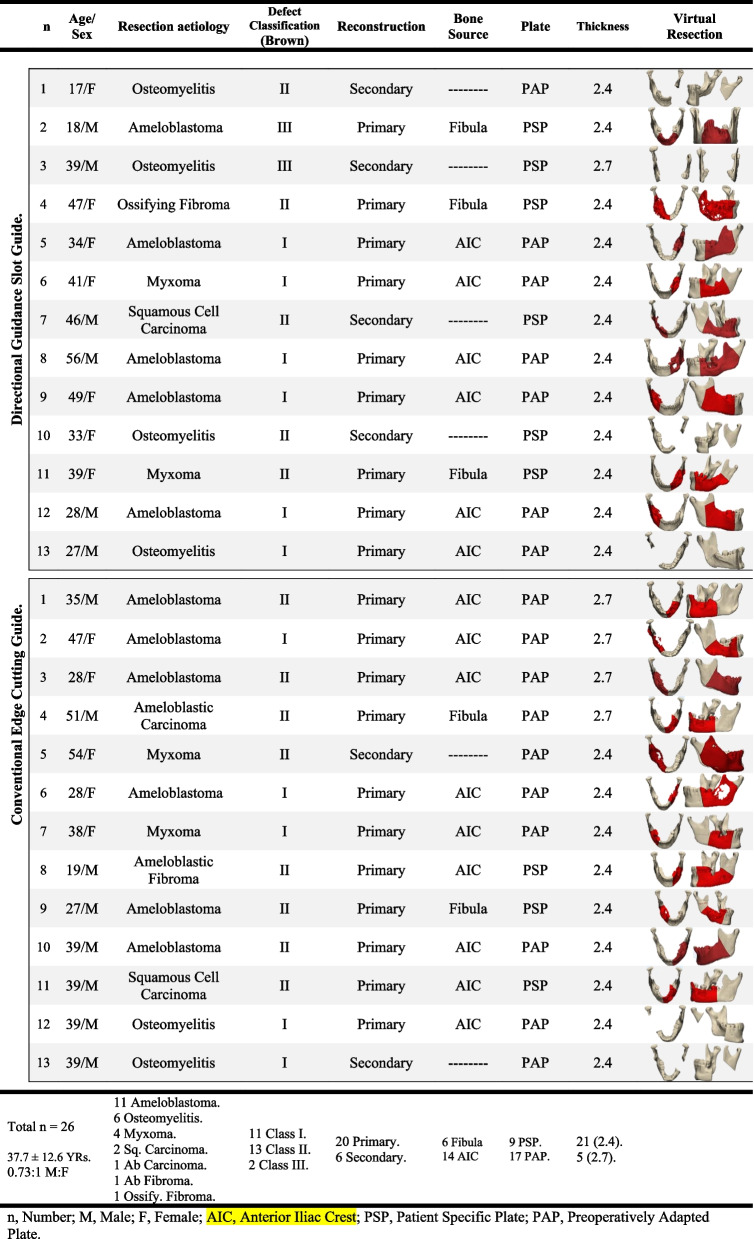
Patients’ demographic data presentation

The absolute mean linear difference reported in the study group was 0.49 ± 0.19 mm for the proximal plane, and 0.30 ± 0.19 mm for the distal one. On the other hand, the control group reported a 2.1 ± 1.2 mm and 2 ± 1.2 mm for the proximal and distal planes respectively. The difference between both groups was statistically significant (*P* < *0.001*). Regarding the pitch plane rotation around the buccolingual axis, the study group reported 1.1 ± 0.61° and 1.3 ± 0.50 ^o^, while the control group reported 2.2 ± 0.55° and 4.7 ± 4^o^ for the proximal and distal planes respectively. In analysis of the yaw plane rotation around the craniocaudal axis, the study group reported 2 ± 0.58° and 2.4 ± 0.33 ^o^, while the control group reported 5.1 ± 3.1° and 9.1 ± 9^o^ for the proximal and distal planes respectively. In both pitch and yaw rotation alterations, the difference between the guide with the positional guidance and the conventional edge-cutting guide was statistically significant (*P* < *0.001*) (Table 2).

**Table 2:** Analysis of the positional accuracy of the resection planes from different resection guide forms (*n* = 26)

		**Proximal Osteotomy**			**Distal Osteotomy**		
		**Distance (mm)**	**Pitch (**°**)**	**Yaw (**°**)**	**Distance (mm)**	**Pitch (**°**)**	**Yaw (**°**)**
**Directional Guidance Slot Guide (*n* = 13)**	*Δ* ± *SD*	0.49 ± 0.19	1.1 ± 0.61	2 ± 0.58	0.30 ± 0.19	1.3 ± 0.50	2.4 ± 0.33
	*Range*	0.21 – 0.81	0.16 – 1.8	1.4 – 3.1	0.11 – 0.65	0.54 – 2	1.9 – 3
**Conventional Edge Cutting Guide (*n* = 13)**	*Δ* ± *SD*	2.1 ± 1.2	2.2 ± 0.55	5.1 ± 3.1	2 ± 1.2	4.7 ± 4	9.1 ± 9
	*Range*	0.67 – 4.2	1.5 – 3.1	2.2 – 13.3	1.2 – 4.8	0.56 – 12.7	2.7 – 33.5
Test (*P*)		U = 4.0^*^ *(*< 0.001^*^)	t = 5.104^*^ *(*< 0.001^*^)	U = 10.0^*^ *(*< 0.001^*^)	U = 0.0^*^ *(*< 0.001^*^)	U = 22.0^*^ *(*< 0.001^*^)	U = 4.0 *(*< 0.001^*^)

*P p* value for comparing between the studied groups

*Δ* Absolute mean, *SD* Standard deviation, *t* Student t-test, *U* Mann Whitney test

^*^Statistically significant at *p* ≤ 0.05

Primary reconstruction was conducted in 20 patients, which was conducted using fibula flap in 6 cases and with Anterior Iliac Crest (AIC) in 14 patients. Insertion Accuracy of the harvested reconstruction bone block in the resected defect in the study group reported a mean lateral shift of 0.74 ± 0.28mm and vertical shift of 0.62 ± 0.24mm in the proximal plane, and 0.72 ± 0.23mm lateral shift, and 0.63 ± 0.33mm vertical shift in the distal plane. Differences in both lateral and vertical shifts in both the proximal and distal planes between the study and the conventional group were statistically significant (*P* < *0.001*) (Table 3).

**Table 3:** Analysis of the flap/graft positional accuracy (*n* = 20)

		**Proximal Osteotomy**		**Distal Osteotomy**	
		**Lateral Shift (mm)**	**Vertical Shift (mm)**	**Lateral Shift (mm)**	**Vertical Shift (mm)**
**Directional Guidance Slot Guide (*n* = 13)**	*Δ* ± *SD*	0.74 ± 0.28	0.62 ± 0.24	0.72 ± 0.23	0.63 ± 0.33
	*Range*	0.24 – 1.2	0.34 – 0.93	0.22 – 1	0.20 – 1.2
**Conventional Edge Cutting Guide (*n* = 13)**	*Δ* ± *SD*	2 ± 1.5	1.2 ± 0.68	2.4 ± 1.3	1.3 ± 0.43
	*Range*	0.69 – 5.2	0.19 – 2.8	0.69 – 4.8	0.76 – 2
Test *(P)*		U = 24.50* *(*< 0.009*)	U = 34.00* *(*< 0.001*)	U = 13.00* *(*< 0.001*)	U = 20.50* *(*< 0.001*)

*P p* value for comparing between the studied groups

*Δ* Absolute mean, *SD* Standard deviation, *t* Student t-test, *U* Mann Whitney test

^*^Statistically significant at *p* ≤ 0.05

In defect extent analysis, the study group reported a mean difference of -0.09 ± 0.37 mm. An excellent level of agreement was reported between the preoperative and postoperative defect span length (*ICC* = *1.00*). This excellent level of agreement was also reported in the control group but with a mean difference of 0.59 ± 1.27 mm (*ICC* = *0.995*) (Table 4).

**Table 4:** Analysis of the defect span accuracy (*n* = 26)

Segment L (mm)	Directional Guidance Slot Guide (*n* = 13)	Edge cutting conventional Guide (*n* = 13)
**VPM**		
Δ ± SD	69.64 ± 22.54	64.7 ± 13.4
Range	48.6 – 120.5	43.7 – 86
**APM**		
Δ ± SD	69.55 ± 22.58	65 ± 13.1
Range	48.7 – 120.3	44.1 – 85.1
**Difference**		
Δ ± SD	-0.09 ± 0.37	0.59 ± 1.27
Range	-0.69 – 0.80	-1.1 – 2
**Level of agreement**		
ICC coefficient	1.000	0.995
95%C.I	1.000 – 1.000	0.982 – 0.998
*P*	< 0.0001*	< 0.0001*

ICC Outcome Values: < 0.5 Poor agreement, 0.5 to < 0.75 Moderate agreement, 0.75 to < 0.9 Good agreement, 0.9—1.0 Excellent agreement

*VPM* Virtual Preoperative Model, *APM* Actual Postoperative Model, *Δ* Absolute mean, *SD* Standard deviation, *ICC* Interclass Correlation Coefficient, *CI* Confidence interval

*P p* value for comparing between the studied groups

^*^ Statistically significant at *p* ≤ 0.05

## Discussion

Resection-osteotomy guides are used to translate the virtual setting into the operation room, for accurate mimicking of the computer-assisted surgery. Accurate reconstruction is interconnected with the accuracy of the resection guide in transferring the three-dimensional position intraoperatively [7, 15]. With the lack of consensus regarding the ideal configuration of the resection guide, this study aimed to compare the accuracy of different resection guides in translating the three-dimensional virtual position intraoperatively.

A total of 52 osteotomies were conducted and analyzed in this study. The positional accuracy of the resection guide was evaluated in a linear deviation manner, by antero-posterior deviation measurement, and in an angular manner, by pitch rotation around the FHP and yaw rotation around the MSP difference calculation. Since the resection was implemented all the way from the buccal to the lingual, roll rotation analysis was of no value. In both groups a satisfactory millimeter linear and three-dimensional positional accuracy was reported, however cases where a resection guide with a directional guidance slot was utilized reported a statistically significant spatial positioning of both resection planes.

Regarding the liner antero-posterior deviation, the difference between the directional-guided and the edge-cutting guides was statistically significant (*P* < *0.001*). The greatest reported mean deviation was 2.1 ± 1.2 mm for the proximal plane in the control group, and the least reported mean deviation was 0.30 ± 0.19 mm for the distal plane in the study group. The resection guide accuracy evaluation is usually reported in the literature in a two-dimensional form analysis, with the presentation of the distance difference between the corresponding resection planes. Roser et al. conducted a retrospective study for the analysis of the accuracy of virtual planning in cases with mandibular resection and fibular graft reconstruction. Their study reported a total of 19 osteotomies, with a mean linear deviation of 2.00 ± 1.12 mm [9]. Shu et al. evaluated the accuracy of CAS in the reconstruction of mandibular defects with iliac crest graft. They documented a mean resection plane deviation of 2.3 ± 1.0 mm [17]. Both of the above-mentioned studies utilized a conventional edge-cutting resection plane, and their results fall in line with those obtained in the control group cases [9, 17]. Brouwer de Koning et al. utilized a resection guide with a directional guidance slot for the conduction of their mandibulectomy procedure [10]. They reported a mean deviation of 2.2 ± 0.9 mm and 1.2 ± 1.0 mm for the posterior and anterior osteotomies respectively [10]. In this study along with that conducted by Brouwer de Koning et al., a lesser value of deviation was reported in the distal plane than in the proximal one [10]. Zho et al. compared the efficacy of two different resection guide forms in mandibular reconstruction using a vascularized iliac crest flap [18]. Their complicated guide contained a directional guidance slot, which reported a mean linear conjunction gap difference of 1.6 ± 0.7 mm [18]. The favourable negatable millimetre deviation reported in this study is correlated with the favourable postoperative resection specimen histopathological analysis, where none of the cases showed a positive margin. Accordingly, one of the main leverages of the VSP in mandibular reconstruction is lowering the rate of positive bone margin.

Changes in the linear dimension may not provide a complete picture regarding the three-dimensional position of the resection plane. Regarding the angular antero-posterior deviation, the templet with a directional guidance slot reported a statistically significant difference in both the pitch and the yaw rotation deviations when compared to the conventional edge-cutting guide (*P* < *0.001*). The greatest reported mean deviation was 9.1 ± 9^o^ for the yaw rotation of the distal plane in the control group, and the least reported mean deviation was 1.1 ± 0.6° for the pitch rotation of the proximal plane in the study group. Brouwer de Koning et al. reported a comparable plane-angular deviation, with a mean anterior osteotomy deviation of 2.6° pitch and 5.1° yaw, and a mean posterior osteotomy deviation of 4.2° pitch and 9.5° yaw [10].

The difference between both groups regarding the yaw angle indicates that the directional guidance slot was able to control the saw movement through the resection procedure, which was not achieved in the control group. Errors in angular deviation usually lead to deviation of the osteotomy toward the resected part, leading to a reduction of the safety margin, and less bone is removed. Furthermore, the impeccable angular deviation outcome in the cases where a guide with a directional slot was utilized demonstrates that the slot and the saw did not have any excess clearances that may allow any unwanted directional deviation. This was attained by preoperative calibration of the utilized oscillating saw blades and designing the slot in accordance with this size and an offset of 0.01. Additionally, Changes in the angulation of the resection plane may provide difficulties in the position of the reconstruction bone block, as the harvested blocks are usually fabricated to the shape of the defect in the virtual setting. Despite that, the reported angular deviation had a minimal effect on the graft-stump contact and implanted bone blocks did not need any manual adjustment, and a precise fit was obtained. However and according to our experience, the cases with the directional guidance slot showed a better fit in the reconstructive bone block insertion, especially when a customized reconstruction plate was utilized.

Despite being both conducted with satisfactory accuracy, the proximal plane showed a more accurate angular performance while the distal plane showed a more accurate linear performance. Brouwer de Koning et al. conclude that seating the guide in the anterior region is an easier procedure since it is better exposed and accessed [10]. Positioning of the proximal osteotomy may be hampered by the soft tissue overlying the ramus and inadequate exposure, which increases the deviation of the cutting tool's 3D direction. This may show the importance of the creation of a directional guidance slot to contain the saw direction throughout the resection procedure. Which once seated, the direction of the cutting will not be affected.

Insertion Accuracy of the harvested reconstruction bone block in the resected defect was analyzed to determine the accuracy of the preoperative VSP and the effect of the resection guide in maintaining the space for accurate reconstruction bone positioning. Both groups reported an excellent level of agreement between the virtual and actual defect extent (*ICC* = *1.00*). Despite that, the guide with a directional guidance slot yielded an absolute mean difference of -0.09 ± 0.37 mm, in comparison to the 0.59 ± 1.27 mm in the conventional group.

The study was aimed at determining the accuracy of the resection template in transferring the three-dimensional resection plane position intraoperatively. Despite that, the study adhered to the guidelines proposed by van Baar et al. and modified by El-Mahallawy et al. in reporting CAS in mandibular reconstruction surgery [7, 15]. Standardization of the preoperative and postoperative MSCT machine and scanning parameters, reporting in Brown classification, overlapping the condylar segments, XYZ planes alignment, and finally statistical analysis with the agreement between virtual and postoperative actual measurements was performed in this study. These guidelines helped in a robust analysis of the resection plane positional accuracy, along with obtaining reproducible outcomes in an attempt for standardization.

In this study, primary reconstruction with implantation of bone graft/flap was conducted in 20 patients. Insertion Accuracy of the harvested reconstruction bone block is imperative in order to achieve an immanent graft-stump contact [9, 10]. The outcome of the resection guide with a directional slot was statistically significant when compared to the conventional edge-cutting guide in both lateral and vertical shifts, and in both the proximal and distal planes. The directional guidance template showed a millimetre accuracy with the least reported absolute mean deviation of 0.62 ± 0.24mm in the proximal plane vertical shift, and heights reported an absolute mean deviation of 0.74 ± 0.28mm in the proximal plane lateral shift. Ciocca et al. analysed the accuracy of fibular graft insertion with the use of a 3D-printed reconstruction plate. They reported a lateral shift of 1.36 and 2.22, and vertical shifts of 2.93 and 2.90 mm for the anterior and posterior block points respectively [19]. The outcomes in this study are comparable and even more accurate, especially in the cases managed with a directional slot resection guide form. This satisfactory clinical performance in graft/flap insertion may be accredited to the utilization of a reconstruction-fixation guide, which utilized the same screw boreholes as the resection guide for three-dimensional spatial control of the area between the proximal and distal stumps during the period of plate fixation. In this study, we utilized the reconstruction-fixation guide in all of the enrolled cases and even in those managed with a patient-specific plate.

Custom-made resection guides are used to translate the virtual blueprint into the actual operation room. The main intent of any rehabilitative procedure is primarily the complete eradication of the diseased tissue with the appropriate safety margin in order to prepare the surgical bed for the reconstruction step [20]. The accuracy of the resection guide insertion and osteotomy performance is directly proportionate to the complete tumour resection and reconstructive procedure. The study is limited by the variability in the manner of inclusion of the mandibulectomy defect, however, it aimed to limit the confounding variables by excluding cases with condylar resection. Furthermore, the assessment of the resection plane and block position accuracy mandates proficiency in several computer programs with a steep learning curve.

Errors in the exact position of the osteotomy guide have an impeding effect on the fitting of the harvested graft, which increases operative time and lessens the accuracy of the procedure [21]. Chackartchi et al. report that a more complex design and achieving more virtual work to produce a totally limiting implant placement guide consequences in a more precise implant positioning with few errors and a decrease in the procedure time [22]. The same goes for the maxillofacial reconstruction field. An improved design technique influences the procedure conveyance with the limitation of free-hand deviations. The use of virtual planning in mandibular reconstruction is of proven accuracy [7, 15]. Despite that, the literature lacks uniformity about a definitive sculpt for the guides as it is normally a surgeon/engineer experience-based with demand-based changes. The utilization of a directional guidance slot in the resection-osteotomy guide provided the surgery with impeccable accuracy and control over the three-dimensional position of the osteotomy and the reconstruction bone block.

The study is limited by the fact that precise limitation of the included cohort sample is difficult which may affect the results of the study as different location of the osteotomy plans may result in different positional difficulty of the resection guide. However, the study took all the measures to avoid this bias and opted for exclusion of cases with joint invasion and subsequent joint prosthesis fabrication. The outcomes reported in this study in both groups documented a satisfactory outcome. This entails the well-proven accuracy of virtual surgical planning in maxillofacial surgery and that the utilization of the contemporary computer-assisted modalities is mandated and applicable.

It could be concluded from the outcome of this study that the conduction of the osteotomy was more proficient when a directional guidance slot was incorporated into the design. It allowed an accurate transfer of the three-dimensional position of the virtual resection plane intraoperatively with satisfactory overall clinical performance. The promising results may endorse the generalization of the resection guide design with a directional guidance slot in computer-assisted mandibular resection procedures.

## Data Availability

All data generated during this study are included in the article, along with the analysis.

## Abbreviations

VSP: Virtual Surgical Planning
3D: Three-Dimensional
CAD/CAM: Computer-Aided Design/ Computer-Aided Manufacturing
CT: Computed Tomography
MDCT: Multi-Detector Computed Tomography
CAS: Computer-Assisted Surgery
DICOM: Digital Imaging and Communications in Medicine
VPM: Virtually Preoperative Model
STL: Standard Tessellation Language
AIC: Anterior Iliac Crest
FDM: Fused Deposition Model.
CDC: Canter for Disease Control
FHP: Frankfurt Horizontal Plane
APM: Actual Postoperative Model
Bpp: Block proximal point
Bdp: Block distal point
MSP: Mid-sagittal Plane
FHP: Frankfurt Horizontal Plane
ICC: Intra Class Correlation Coefficient
